# Effects of influenza A infection on liberation of bacteria from biofilms and inflammatory response in an in vitro model of chronic rhinosinusitis

**DOI:** 10.1099/mic.0.001586

**Published:** 2025-08-11

**Authors:** Jordan Hall, Alkis Psaltis, Sarah Vreugde, Sholeh Feizi, Yimin Chuah, Mohammed Alsharifi, Mahnaz Ramezanpour

**Affiliations:** 1 The University of Adelaide, Adelaide, Australia; 2 The Queen Elizabeth Hospital, Adelaide, Australia

**Keywords:** chronic rhinosinusitis, influenza A, IL-6, *Pseudomonas aeruginosa*, pyocyanin, pyoverdin

## Abstract

Chronic rhinosinusitis (CRS) is a prevalent and life-altering disease characterized by the persistent inflammation of the sinuses lasting longer than 3 months. *Pseudomonas aeruginosa* (PA) is a prominent biofilm-forming bacterium that colonizes the sinuses of up to 9% of CRS patients. PA in biofilm exhibits a great resistance to antibiotics and has proven difficult to remove from the sinus mucosa of CRS patients. Influenza A virus (IAV) is the second most common virus detected colonizing CRS patients’ sinuses, with previous studies finding that IAV-induced inflammation of human cells causes the dispersal of bacteria encased in biofilms, leading to increased disease exacerbations. Yet, the two pathogens and the effect that co-infection with them has on primary human nasal epithelial cells (HNECs) have to be assessed. In this study, we show that co-infection of HNECs with both clinical and laboratory isolates of PA and with IAV causes no significant change in PA biofilm biomass and no significant change in the production of PA virulence factors. We also show that co-infected HNECs exhibit lower IL-6 response when compared to HNECs infected with IAV alone, suggesting a novel finding where PA is dampening IL-6 response once co-infection occurs.

## Data availability

All relevant data and resources can be found within the article and its supplementary information.

## Introduction

Chronic rhinosinusitis (CRS) is defined as inflammation of the sinuses persisting longer than 3 months despite antibiotic interventions [1]. CRS can be grouped into two main clinical phenotypes, CRS with nasal polyps (CRSwNP) and CRS without nasal polyps (CRSsNP) [2]. The Australian Institute of Health and Welfare reports that CRS has a prevalence of 8% in the Australian population and is associated with a lower quality of life and increased financial burden [3]. CRS has three primary risk factors: genetic predisposition, comorbidities with other inflammatory diseases and environmental factors [2]. Possible environmental triggers for CRS comprise factors such as the presence of bacterial biofilms and associated infections, fungal infections, allergies, smoking and exposure to environmental pollutants [4]. Common pathogens in the upper respiratory tract include *Staphylococcus aureus* and *Pseudomonas aeruginosa* (PA). PA is regularly detected in the sinonasal cavities of CRS patients and is linked to severe recalcitrant CRS [5]. PA, a life-threatening Gram-negative bacterium, is currently prioritized by the World Health Organization for antibiotic research due to rising resistance and prevalence [6,7]. PA not only colonizes CRS patients but is also inextricably linked with cystic fibrosis (CF), with the pathogen being the predominant microbe in 70% of CF patients [8]. PA serves as a model organism for studying biofilm formation, as well as virulence factors like pyocyanin and pyoverdin, due to its common occurrence [9]. A study conducted by Tuli *et al*. found that exoproteins from both planktonic and biofilm PA isolates induced increased IL-6 production in airway epithelial cells, causing damage to the mucosal membrane and associated with exacerbation of CRS symptoms [10]. Identification of biofilms in patients with CRS is associated with a poorer prognosis as well as a lower quality of life [2]. Biofilm dispersal, the release of bacterial cells from established biofilms, is a complex process due to cells being cemented in an extracellular polymeric substance matrix. Despite its potential impact on biofilm development and patient prognosis, dispersal effects remain inadequately studied. Recent studies have shown that viral infections can induce the release of virulent bacteria from established biofilms [11,13]. Influenza (IV) commonly colofnizes CRS patients’ nasal epithelia and is a virus known to cause the dispersal of planktonic bacteria from its biofilm form [11]. IV is an acute respiratory virus caused by a negative-strand RNA virus of the *Orthomyxoviridae* family of which there are four genetically distinct species: Influenza A virus (IAV), Influenza B virus (IBV), Infleunza C virus (ICV) and Influenza D virus (IDV). IAV strains can cause pandemics due to their zoonotic ability as well as their susceptibility to antigenic variation [14]. IBV strains have also been known to cause seasonal epidemics but are detected at a far lower rate than IAV strains and are not responsible for pandemics [15]. ICV infections generally cause mild illness and are not responsible for epidemics or pandemics but may be a threat to people with compromised immune systems [15]. IDV strains primarily affect cattle and swine with their pathogenesis and impact on human health being relatively unknown [16]. Interestingly, IAV has been shown to cause the dispersal of bacterial biofilms, due to IAV-induced increases in norepinephrine, ATP, glucose and the lysis of cells. A study conducted by Marks *et al*. confirmed that the resulting host signals of IAV virus-infected human lung epithelial cells caused the dispersal of *Streptococcus pneumoniae* from its biofilm form. When exogenously applying norepinephrine, ATP, glucose or cell lysates, a significant and rapid decrease of bacteria from within attached biofilm communities as well as an increase in bacterial cells in supernatant was observed. Similarly, simulation of a feverish temperature (>37 ℃) showed equivalent results, with the effect being most predominant at 38.5 ℃ [12]. Furthermore, IAV virus infection is intrinsically associated with the dissemination of pneumococci from the nasopharynx to the middle ear, contributing to the onset of otitis media [12]. These findings indicated that infection with the IAV virus caused an increase in the virulence factors of bacteria present [12,13]. Another study by Pettigrew *et al*. found that actively dispersed *Streptococcus pneumoniae* were more virulent due to an increase in metabolic activity as well as through upregulation of genes associated with the production of bacteriocins and downregulation of genes associated with colonization [17]. The interactions between viruses and bacterial biofilms have yet to be studied extensively, with studies only considering the interaction between other commonly occurring viruses and bacteria such as rhinoviruses or *Streptococcus pneumoniae* present in patient nasal and respiratory cavities [12,17]. Many factors of other viral and bacterial co-infections have been looked at, such as the gene alterations that occur in both bacteria and respiratory cells, the inflammatory response that occurs and the mechanism behind the dispersal of bacteria from biofilm [16]. However, previous studies have failed to consider what the interaction between IAV and PA biofilm is in CRS patient human nasal epithelial cells (HNECs) when co-infection has occurred.

This study investigates whether co-infection of HNEC cultures with PA and IAV leads to biofilm dispersal, increased cytokine levels and upregulated PA virulence factors.

## Methods

### Reagents

PneumaCult™ Ex-Plus Culture media was purchased (STEMCELL Technologies Australia Pty. Ltd, Tullamarine, VIC, Australia). Human blood was provided by consenting patients from the Queen Elizabeth Hospital.

### Viral strains

Influenza A virus (A/California/07/2009(H1N1)) was propagated in 10-day-old embryonated hen eggs (by Dr. Mohammed Alsharifi at the School of Biological Sciences, The University of Adelaide). IAV was titrated by a 50% tissue culture infectious dose (TCID_50_) assay using HNECs. HNEC cells were maintained in complete Ex-plus and 1% penicillin/streptomycin. HNECs were kept at 37 °C with 5% CO_2_ and were passaged with trypsin when they reached ~90% confluence. For the TCID_50_ assay, HNECs were seeded in 96-well round-bottomed plates at 1.5×10^4^ cells well^−1^. After 24-h incubation, confluent cell monolayers were infected with tenfold serial dilutions of IAV in complete Ex-plus supplemented with 1% FBS and 8% trypsin for virus activation. Plates were incubated at 37 °C for 3 days, and then amplified virus in culture supernatants was detected by the addition of 0.6% packed red blood cells (RBCs) based on pellet or mesh formation, with a mesh being considered positive for IAV. 50% infectious doses (TCID_50_ ml^−1^) were calculated using the Reed and Muench method [18].

### Bacterial strains/biofilm formation

The isolates were acquired from the sputum of a patient with cystic fibrosis (C10) and one without cystic fibrosis (7596). The identification of the isolates as *P. aeruginosa* (C10 and 7596) was carried out utilizing MALDI-TOF MS (Bruker MBT, SA Pathology, Adelaide, Australia). All isolates used were assessed for their mucoidal status by plating the bacterial strains on Congo red agar (see Fig. S3, available in the online Supplementary Material); deep red colony formation indicates mucoidal status, whereas white/pink colony formation indicates a non-mucoidal status. For experiments, clinical isolates were grown on nutrient broth agar plates for 24 h and then suspended in 0.9% saline at 1±0.1 McFarland units. Isolate suspension was then added to tryptic soy broth (Sigma-Aldrich Australia Pty. Ltd, Bayswater, VIC, Australia) and grown on a 96-well in a pegged plate (Innovotech, Edmonton, Canada); plates were left to incubate at 37 ℃ for 48 h on a rotator plate to form biofilm.

### Human nasal epithelial cell culture

Primary HNECs were gently harvested from the surface of the polyps for CRSwNP cases and from the middle turbinates for non-CRS controls using sterile nasal brushes. Nasal brushings were then suspended in Dulbecco’s Modified Eagle Medium (Thermo Fisher Scientific, Adelaide, Australia). The extracted cells underwent a depletion process to remove monocytes by utilizing anti-CD68 (Dako, Glostrup, Denmark)-coated culture dishes as described previously and then grown in PneumaCult™ Ex-Plus culture media [19].

### Biofilm dispersal assay

HNEC cells were cultured until 80% confluent and then harvested for seeding onto collagen-coated 96-well plates (Greiner Bio-One, Kremsmünster, Austria) at a density of 15,000 cells per well. The next day, HNECs were incubated with IAV at a multiplicity of infection (MOI) of 1 for 24 h at 37 °C in a CO2 incubator. The following day, the pegs (used for biofilm formation) were rinsed with sterile PBS three times, and the pegs were immersed in the 96-well plate containing HNECs infected with the IAV virus for 3 h. Subsequently, pegs were washed twice with PBS to remove bacteria released from the biofilm, stained with 0.1% crystal violet and left to dry overnight. The bound dye was eluted with 180 µl of 33% acetic acid, and the OD was measured at a wavelength of 595 nm to quantify the biofilm mass.

### Assessing extracellular infection

Supernatant was collected from both infected and control cells (uninfected). Supernatant was serially diluted in PBS; the serial dilutions were then added to cetrimide agar plates. Plates were incubated for 24 h at 37 ℃, and c.f.u. per millilitre was enumerated for each bacterial strain in different conditions.

### Assessing the upregulation of pyoverdin

To assess pyoverdin levels, 20 µl of the supernatant containing bacterial cells of both IAV-infected and non-IAV-infected cells was added to cetrimide agar plates and incubated overnight at 37 ℃. Note that assessment of the virulence factors of PA exposed to IAV alone was not conducted, as previous studies have shown that the upregulation of virulence factors is due to IAV-induced cell-mediated changes rather than direct interactions between the IAV and PA themselves [17]. Visual assessment was then done to determine the release of pyoverdin by PA colonies. Further assessment of pyoverdin release was conducted by growing PA isolates on Luria–Bertani (LB) agar overnight, then inoculating a loopful of bacteria into an LB liquid culture for 24 h. One hundred microlitres of liquid culture were transferred to a 96-well plate, and pyoverdin fluorescence (Ex. 405 nm; Em. 460 nm) was measured using a CLARIOstar Plus microplate reader (BMG Labtech, Offenburg, Germany) [20].

### Assessing the upregulation of pyocyanin

To assess pyocyanin levels, 20 µl of the supernatant of both IAV-infected and non-IAV-infected cells was added to skim milk agar and incubated overnight at 37 ℃. Note that assessment of the virulence factors of PA exposed to IAV alone was not conducted, as previous studies have shown that the upregulation of virulence factors is due to IAV-induced cell-mediated changes rather than direct interactions between the IAV and PA themselves [17]. Visual assessment of PA growth was then done to determine virulence factor production. Further assessment of pyocyanin was conducted as described previously [21]. In short, PA isolates were grown on nutrient agar overnight, and then a loopful of bacteria was inoculated into a nutrient broth liquid culture for 24 h to allow for production of the metabolite. The liquid culture was then centrifuged at 4,355 ***g*** for 15 min, and supernatants were collected. 4.5 ml of Chloroform was added to the supernatant, and the mixture was vortexed for 20 s. Mixtures were then centrifuged further at 4,355 ***g*** for 10 min, chloroform was transferred to a separate tube and 1.5 ml of 0.2 N HCL was added to the chloroform, mixed and centrifuged for a further 10 min. HCL on top of the chloroform is used to measure absorbance (Ex. 405 nm; Em. 460 nm) using a CLARIOstar Plus microplate reader (BMG Labtech).

### IL-6 ELISA

Supernatant collected from HNECs was used to determine IL-6 levels. Biotin rat anti-human IL-6 (BD Biosciences, NJ, USA) was used as the detection antibody, and purified rat anti-human IL-6 (BD Biosciences) was used as the capture antibody. Absorbance data were measured using FLUOstar OPTIMA (BMG LABTECH). Absorbance data and standard curve were analysed using OPTIMA MARS software.

### Statistical analysis

Statistical analysis of all results was conducted using GraphPad Prism (version 10.3.0). The ANOVA and T-tests were done to determine the significance of all relevant data sets. HNECs from CRS and non-CRS patients were not statistically analysed separately as there were no differences observed between the two conditions.

## Results

### Determination of IAV replication and concentration in HNECs

We first performed a TCID50 assay, which yielded a titre of 9.54×10⁵. This allowed us to calculate the appropriate MOI for subsequent experiments (MOI=1). While HNECs are not traditionally used for robust IAV propagation like MDCK cells, our preliminary experiments demonstrated that HNECs can support productive infection [22].

### Co-infection of primary human nasal epithelial cells shows no effect on bacterial biofilm biomass

**Fig. 1.**
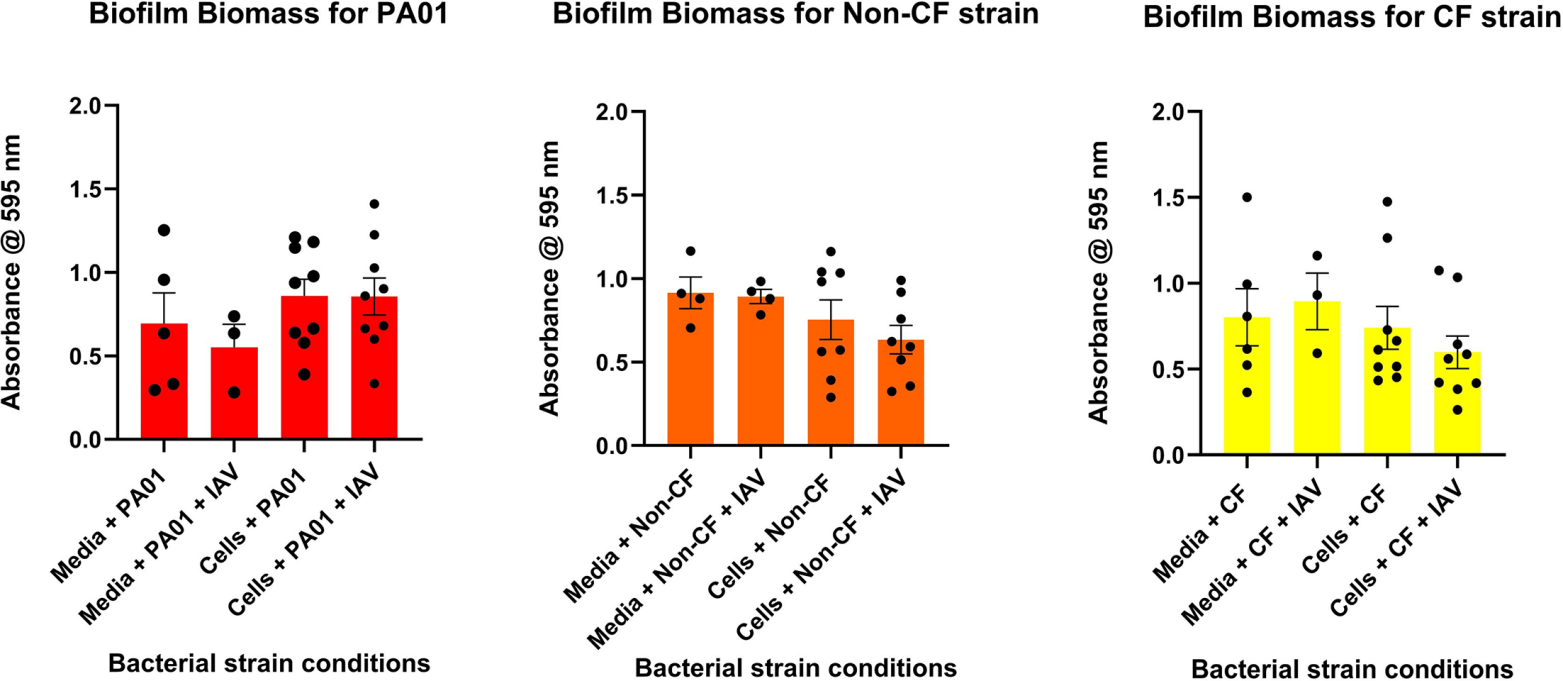
Role of IAV in *P. aeruginosa* biofilm dispersal. PA01 (**a**), non-CF (**b**) and CF (**c**) biofilms were exposed to IAV (MOI-1) at 37 °C for 3 h in the presence or absence of HNECs. The biofilm biomass compared to clinical isolates of PA exposed to media, media+IAV and cells. Data are expressed as mean+sem. Each dot represents 1 replicate; *N*=9 for experimental conditions; *N*=3–4 for control.

We first wanted to evaluate whether IAV in the presence and absence of HNECs affected the biofilm biomass of PA. As shown in Fig. 1, clinical isolates of PA (non-CF and CF) as well as the laboratory isolate PA01 exposed to IAV, in the presence and absence of HNECs, exhibited no significant change in the biofilm biomass compared to the control in any of the conditions tested (*P*>0.05 for all conditions compared to media control).

### PA isolates exposed to IAV lead to no significant change in extracellular PA infectivity and PA production of pyoverdin and pyocyanin

**Fig. 2.**
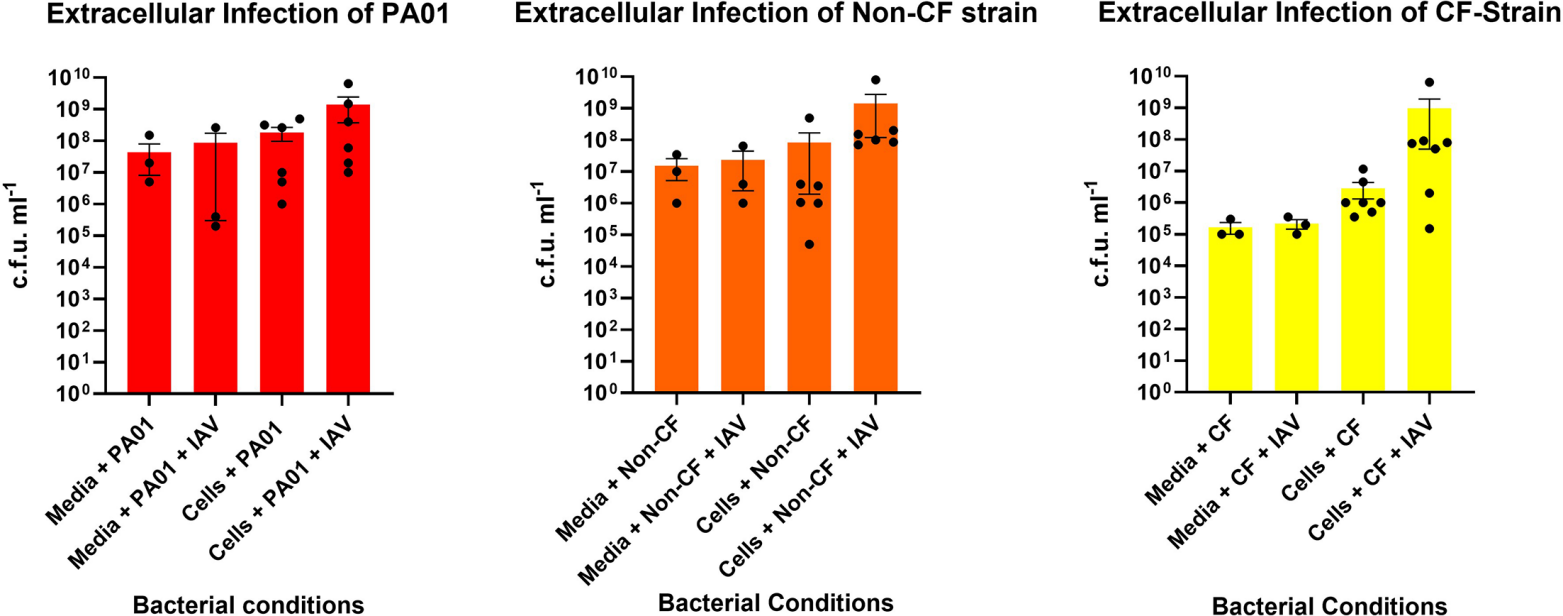
Evaluation of extracellular bacteria. The extracellular bacterial count of PAO1 (**a**) compared to non-CF PA (**b**) and CF PA (**c**) once they are exposed to media, media+IAV, cells and cells+virus. Media conditions acted as controls for the experiment; data are expressed as mean+sem. *N*=5.

**Fig. 3.**
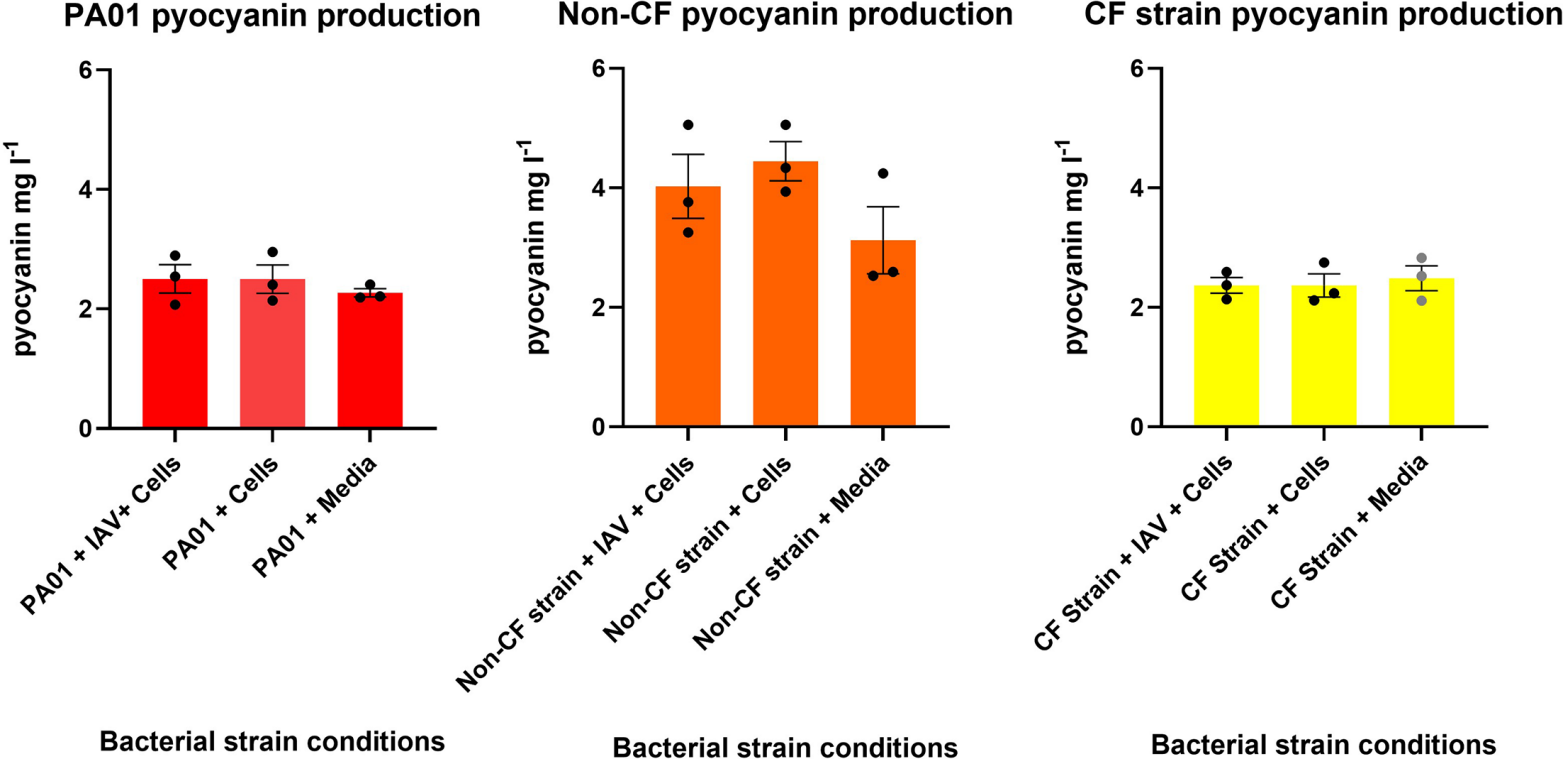
Bacteria virulence factor analysis of pyocyanin production in co-infected cells with IAV and PA. The pyocyanin production in PA01, non-CF and CF strains exposed to different conditions was measured (mg l^−1^). *N*=3; each data point represents four technical replicates averaged.

**Fig. 4.**
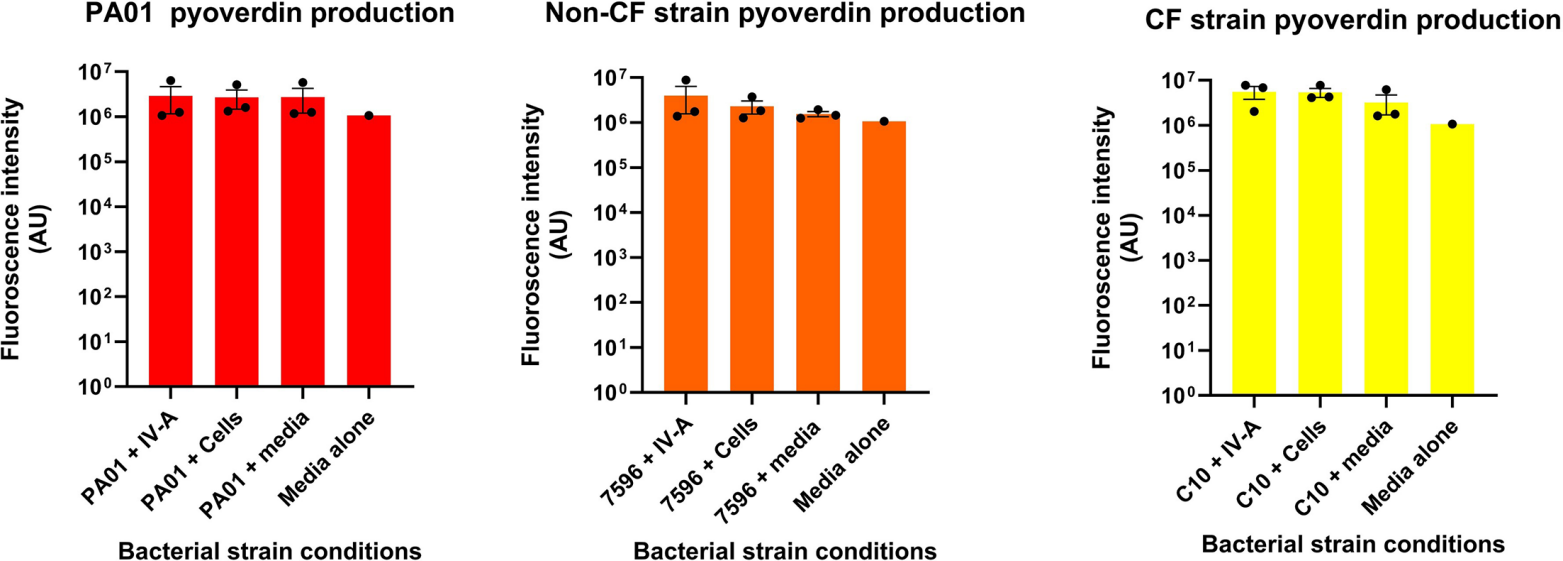
Bacteria virulence factor analysis of pyoverdin production in co-infected cells with IAV and PA. The pyoverdin production in PA01, non-CF and CF strains exposed to different conditions was measured, and fluorescence intensity was measured. *N*=3; each data point represents four technical replicates averaged.

Biofilm dispersal was evaluated by determining the number of free-floating bacteria in biofilm supernatants. As shown in Fig. 2, all bacterial isolates had no significant change in the number of bacteria present in the supernatant after co-infection with IAV had occurred in the presence or absence of HNECs. Co-infection of HNECs with IAV and PA isolates suggested an increase in bacterial virulence factor in all isolates. Fig. S1 shows that in skim milk agar plates, all PA isolates exposed to IAV indicated increased virulence when compared to their non-IAV-exposed counterparts through either an increase in casein hydrolysis or an increased bacterial colony amount. Similar trends were seen in Fig. S2, as PA isolates plated on cetrimide agar with IAV-exposed PA isolates, indicating an increase in virulence through greater fluorescence or an increased bacterial colony amount. Further analysis of the production of virulence factors of both pyocyanin (Fig. 3) and pyoverdin (Fig. 4) shows a stark contrast to the visual assessment of the isolates, with neither result showing that there is a significant difference in the production of these virulence factors (for pyoverdin *P*=0.99, 0.56 and 0.60 and for pyocyanin *P*=0.63, 0.41 and 0.85, respectively) when compared to control conditions. Briefly, visual assessment of PA isolates plated on skim milk and cetrimide agar indicated that increases in pyocyanin and pyoverdin were present; however, quantitative measurements of those virulence factors proved that there is no significant difference.

### Co-infection of cells leads to no significant IL-6 response from HNECSs

**Fig. 5.**
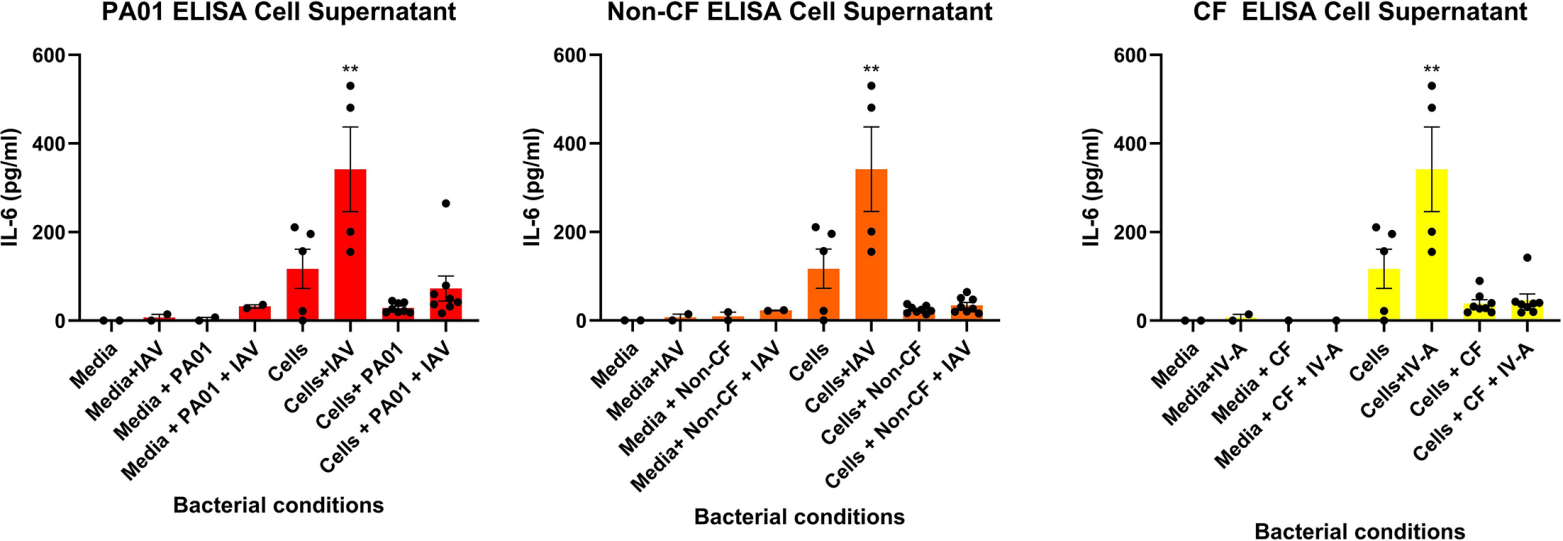
IL-6 secretion of HNEC cultures. Co-infection of cells with IAV and PA isolates including PAO1 (**a**), non-CF PA (**b**) and CF PA (**c**). Media conditions as well as cells alone acted as controls for the experiment; data are expressed as mean+sem. *N*=9 for experimental conditions; *N*=2–4 for control conditions.

IL-6 levels were assessed to determine the inflammatory response in IAV-infected cells and those co-infected with IAV+PA or PA alone. As shown in Fig. 5, cells infected with IAV alone had a significantly higher IL-6 release compared to control cells (*P*<0.05). PA biofilms did not induce IL-6 secretion, and IL-6 secretion was similar in HNECs infected with PA alone or co-infected with IAV and PA (*P*>0.05 compared to control).

## Discussion

Previous studies have shown that biofilm biomass is reduced in multiple different bacteria in the presence of cells when a viral infection has occurred in localized tissue [11,13]. This study challenges that trend with clinical isolates of PA exhibiting no decreased biofilm biomass when in the presence of cells infected with IAV. While the data do show no change in biomass after 3 h of exposure, it is possible that, given enough time, biofilm biomass would decrease when compared to controls. The 3 h time point for incubating biofilm in the presence of cells infected with IAV was chosen primarily because PA is notoriously cytotoxic to cells [23]. A study conducted by Apodaca G. assessed the interaction between PA and MDCK cells, finding that 3 h post-infection PA rapidly caused host cell death and loss of the integrity of the monolayer. Furthermore, the study used a laboratory-grown isolate of PA (PA103), which has previously been shown to be less effective when compared to clinical isolates [23]. While host cell death is a necessary step in assessing biofilm biomass integrity, complete death of all host cells in the monolayer would not be ideal for being able to conduct further experiments on the cells. Another factor to note is that MDCK cells are considered more resilient to external factors when compared to nasal epithelial cells due to their innate immunity responses as well as their ability to form a strong monolayer [24]. The decision to incubate at the chosen time point was further influenced by the fact that cells were not only exposed to PA but were also exposed to IAV 24 h pre-co-infection, as it is well established that viruses cause cell death as a means of replication and dissemination [25]. With no significant decrease in biofilm biomass, one would expect that there would be no increase in the bacteria found in the supernatant. In both clinical and laboratory-grown isolates of PA, no significant increase in the number of bacterial cells in the supernatant can be seen in isolates exposed to IAV-infected cells. This is an expected occurrence given previous data obtained from biomass measurements.

Proteolytic ability refers to a bacterium’s capacity to break down proteins into smaller polypeptides and amino acids, facilitating digestion. A bacterium with higher proteolytic ability *in vivo* could lead to increased breakdown of essential proteins in cellular components, such as membranes and channels [26]. To gain a better understanding of which isolate was more virulent once exposure to IAV had occurred, the proteolytic ability and pyoverdin production of each isolate were assessed by putting collected supernatant onto skim milk agar and cetrimide agar. While both PA01 and the non-CF isolate showed great casein digestion, the CF isolate exhibited a lack of a greenish-blue hue, indicating that this PA isolate lacked the metabolite pyocyanin. A previous study conducted by Nowroozi, Akhavan Sepahi and Rashnonejad found that, in their study population, 95% of their clinical isolates produced pyocyanin and 100% of their isolates gathered from the environment produced pyocyanin [27]. Previous studies highlight the importance of pyocyanin in cystic fibrosis PA, demonstrating that mouse lungs chronically exposed to pyocyanin develop some of the important pathological features that resemble human cystic fibrosis patients chronically infected with PA [28]. Another study assessing the difference in mortality rate of mice infected with a PA strain that produces a high amount of pyocyanin showed a mortality of 100% after 24 h compared to mice who were infected with a standard PA isolate that only showed 66% mortality after 96 h [29]. Further assessment of the metabolite pyocyanin showed that production of pyocyanin was very low, being on average around 4–5 mg l^−1^ across all isolates. This finding is surprising as a study conducted by Shouman H. *et al*. found that when testing a large amount of both clinical and environmental PA isolates, measurements of pyocyanin equating to <5 µg ml^−1^ (or 5 mg l^−1^) were considered low-level pyocyanin production [30]. This shows that despite what visual assessment of the isolates might indicate, there was a below-average production of pyocyanin across the board for all isolates.

To further assess the virulence factor of IAV-exposed bacteria compared to non-IAV-exposed bacteria, isolates were grown on cetrimide agar plates to assess their pyoverdin production [31]. Pyoverdin production acts as a good measure of virulence factor as pyoverdin itself acts as a siderophore, which are bacterial products that bind iron and increase the rate of bacterial iron transport [32]. Visual assessment of all isolates in either IAV-exposed or non-IAV-exposed forms on cetrimide agar plates indicates that bacteria exposed to IAV had an increased virulence factor production. Further quantitative assessment of the metabolite pyoverdin showed that none of the bacterial isolates in co-infection conditions exhibited any significant change in pyoverdin production when compared to control conditions. In a previous study, it was shown that pyoverdin production of 69 PA isolates obtained from CF patients was at the high end around 1×10^6^ arbitrary units (AU) and at the low end of 0, indicating that there is a massive range in the amount of pyoverdin individual isolates can produce [33]. The range of AU that the isolates of this study produced was similar in range to that study, with isolates at a low end producing AU similar to the AU produced by media (1×10^6^), but at a high end producing around 7×10^6^, although this difference was not significant.

Assessing the IL-6 inflammatory response in the supernatant of infected cells acts as a good indicator of whether or not co-infection will lead to an increased inflammatory response [34]. ELISA analysis of IL-6 contained in the supernatant indicated that co-infection of cells with both laboratory and clinical isolates of PA would lead to no significant change in inflammatory response compared to cells infected with the bacterial isolates alone. However, cells infected with IAV alone exhibit the greatest IL-6 response. The effect that IAV has on the inflammatory response in humans is well documented, specifically IL-6 is crucial in the resolution of IAV infection [33]. In a study conducted by Dienz *et al*., it was shown that infection with the H1N1 influenza virus caused a dramatic increase in the levels of pulmonary IL-6 found in infected lungs of mice, similar to what is seen in human lungs, with this increase being deemed as protective and essential for viral clearance [35]. However, this dampening effect that PA seems to have had on IL-6 response when co-infection has occurred is unexpected. A study conducted by Phuong *et al*. found that early PA isolates collected specifically from cystic fibrosis patients would cause a lower expression of cytokines such as IL-6 and IL-8, whereas chronic isolates of cystic fibrosis PA would have the exact opposite effect [36]. Considering that the cells were only exposed to the bacteria for 3 h, the PA isolates would be considered as early infections rather than chronic, explaining why the IL-6 response was low in HNECs infected with PA isolates alone. Low IL-6 response in co-infected HNECs seems to be a novel finding of the study, considering the number of replicates that exhibited a similar result. Cellular death can also not be the cause of this low response, as HNECs were still infected with IAV for the same period in both co-infected and HNECs infected with IAV alone, meaning that extracellular IL-6 should have been at the very least similar between the two conditions, as it is unlikely that a 3-h period contributed to that great of an increase in IL-6 response. A study conducted by Jie *et al*. found that secondary infection of PA post-influenza infection caused an increased IL-6 response 24 h after lung tissue had been infected with PA compared to when tissue was infected with PA or influenza alone [37]. Another study by Chattoraj *et al*. found that mucoid PA dispersed from biofilms in CF patients’ airway epithelial cells increased IL-8 response compared to cells infected with mucoid PA or rhinovirus alone [14]. Both these studies highlight why IL-6 co-infection results obtained from this study should be increased compared to HNECs infected with IAV or PA alone. However, one possible explanation for the lack of elevated IL-6 could be protease-mediated degradation of IL-6 or suppression of its production due to virus-mediated modulation of the host response. A study by Endres *et al*. found that non-mucoid PA isolates can modulate innate antiviral responses of respiratory epithelial cells infected with Rhinovirus [38]. This hypothesis is supported by our findings, as all *P. aeruginosa* isolates used in this study –PAO1, the non-cystic fibrosis isolate and the cystic fibrosis isolate – exhibited a non-mucoid phenotype, confirmed by their whitish coloration on Congo red agar.

To summarize, this study sought to assess whether co-infection of HNECs with both clinical and laboratory-grown isolates of PA and IAV would reduce PA biofilm biomass, increase PA virulence factors and alter IL-6 response in HNECs. We determined that co-infection of HNECs caused no significant decrease in PA biofilm biomass; this was further supported by the absence of a significant increase in PA recovered from the supernatant of co-infected HNECs. Additionally, the PA virulence factors pyoverdin and pyocyanin did not exhibit any significant increase in the presence of IAV-infected HNECs. Notably, IL-6 response in co-infected HNECs was significantly diminished compared to cells infected with IAV alone, suggesting that our PA isolates may be capable of degrading both baseline and IAV-induced IL-6 production.

Although these findings provide insight into PA and IAV co-infection dynamics, a limitation of this study is the absence of a positive control cell line, such as MDCKs, to directly confirm IAV infection efficiency as well as the addition of a positive control of a known biofilm dispersal agent, such as nitric oxide. Including such controls in future experiments would strengthen the interpretation of viral infectivity and help validate host–pathogen interactions more robustly. Future studies should further explore co-infection conditions, with attention to additional virulence factors and inflammatory markers that may be modulated by PA during viral infection.

## Supplementary material

10.1099/mic.0.001586 Uncited Supplementary Material 1.

## Abbreviations

AU: arbitrary units
CF: cystic fibrosis
CRS: chronic rhinosinusitis
HNECs: human nasal epithelial cells
IAV: influenza A virus
IBV: influenza B virus
ICV: infleunza C virus
IDV: influenza D virus
LB: Luria–Bertani
MOI: multiplicity of infection
PA: *Pseudomonas aeruginosa*
TCID_50_: 50% tissue culture infectious dose
