# Supplementary material for: Effects of influenza A infection on liberation of bacteria from biofilms and inflammatory response in an in vitro model of chronic rhinosinusitis
Source: Microbiology (Reading). 2025 Aug 11;171(8):001586. doi: 10.1099/mic.0.001586 (PMC12453117; doi:10.1099/mic.0.001586)
Supplement: Uncited Supplementary Material 1. [file mic-171-01586-s001.pdf]

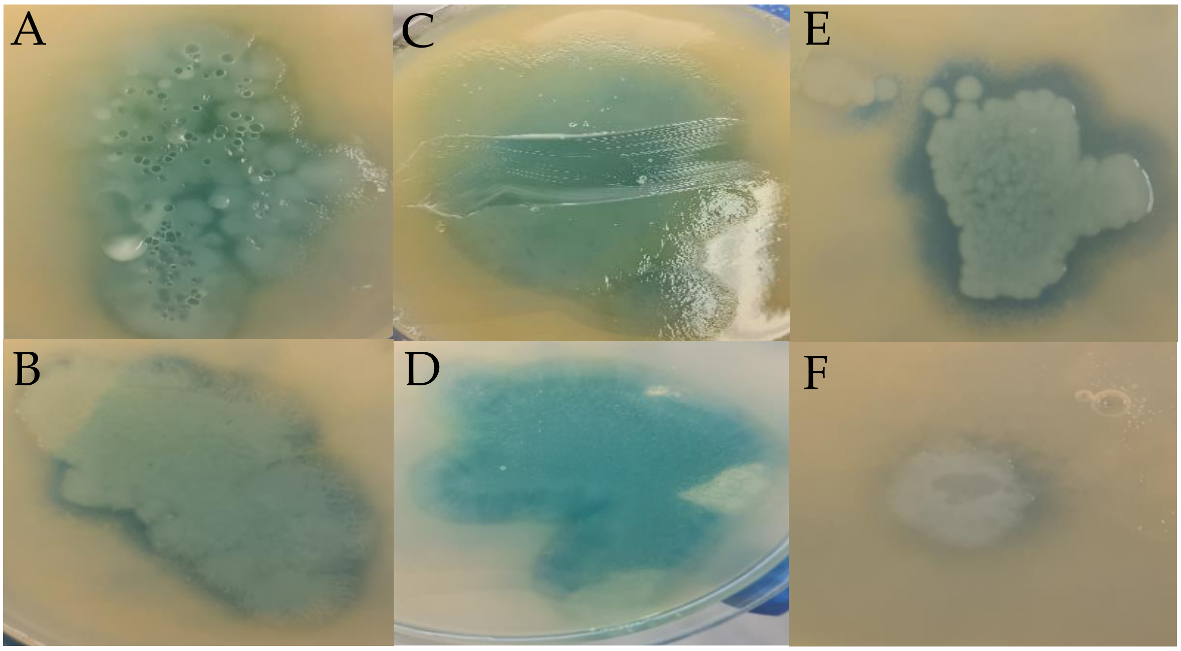

Supplementary figure 1: Bacteria virulence factor analysis of co-infected cells with IAV and PA on skim milk agar. *IAV exposed PA01 (A), Non-CF PA (C) and CF PA (E) and their non-IAV exposed counterpart (B, D and F, respectively). IAV exposed isolates indicate a potential increase in virulence as signified by their increased size or agar degradation. 4 replicates were swabbed, all replicates showed similar results, clearest images were used. IAV: influenza virus-A; PA: Pseudomonas aeruginosa; CF: cystic fibrosis.*

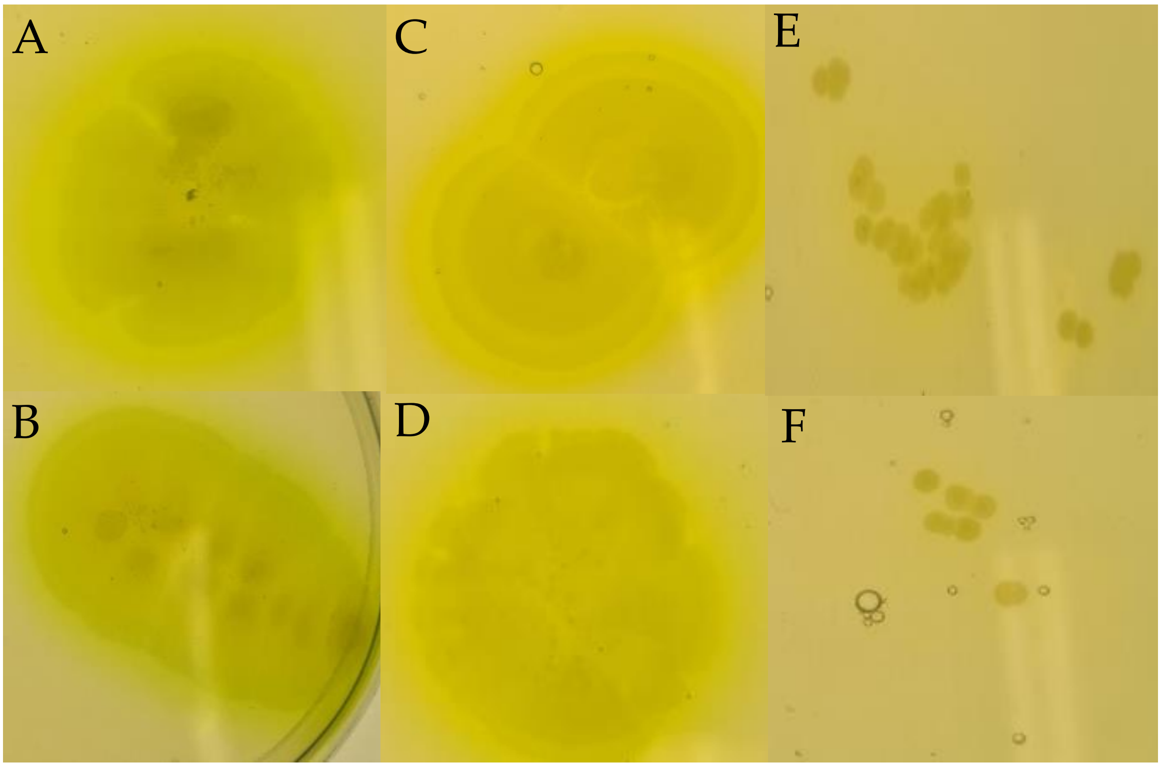

Supplementary Figure 2: Bacteria virulence factor analysis of co-infected cells with IAV and PA on cetrimide agar*. IAV exposed PA01 (A), Non-CF PA (C) and CF PA (E) and their non IAV exposed counterpart (B, D and F, respectively). IAV exposed isolaes indicate an increase in virulence as signified by either their increased quanitity or change in colour. 4 replicates were swabbed, all replicates showed similar results, clearest images were use d. IAV: influenza virus-A; PA: Pseudomonas aeruginosa; CF: cystic fibrosis.*

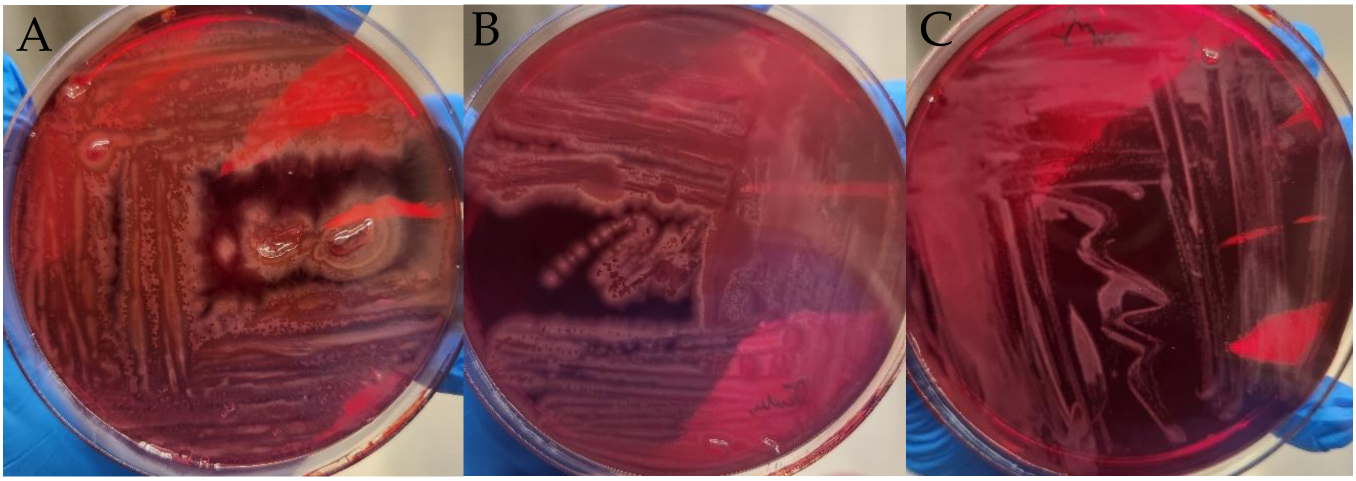

Supplementary figure 3: Bacterial strain analysis for mucoidal isolates. *PA01 (A), Non-CF PA (B) and CF PA (C) growth on Congo red agar indicates that all isolates are non-mucoidal as signified by the whitish colouration of the isolates.*
